# Processing Speed is Related to the General Psychopathology Factor in Youth

**DOI:** 10.1007/s10802-023-01049-w

**Published:** 2023-04-22

**Authors:** Eliza Kramer, Erik G. Willcutt, Robin L. Peterson, Bruce F. Pennington, Lauren M. McGrath

**Affiliations:** 1 University of Denver, Department of Psychology, CO, Denver, US; 2 University of Colorado Boulder, Department of Psychology and Neuroscience, CO, Boulder, US; 3 University of Colorado Boulder, Institute for Behavioral Genetics, CO, Boulder, US; 4 University of Colorado School of Medicine, Aurora, Colorado, US

**Keywords:** *p* factor, General psychopathology, Processing speed, Youth, Structural equation modeling

## Abstract

The relationship between the *p* factor and cognition in youth has largely focused on general cognition (IQ) and executive functions (EF). Another cognitive construct, processing speed (PS), is dissociable from IQ and EF, but has received less research attention despite being related to many different mental health symptoms. The present sample included 795 youth, ages 11–16 from the Colorado Learning Disabilities Research Center (CLDRC) sample. Confirmatory factor analyses tested multiple *p* factor models, with the primary model being a second-order, multi-reporter *p* factor. We then tested the correlation between the *p* factor and a latent PS factor. There was a significant, negative correlation between the *p* factor and PS (*r*(87) = −0.42, *p* < .001), indicating that slower processing speed is associated with higher general mental health symptoms. This association is stronger than previously reported associations with IQ or EF. This finding was robust across models that used different raters (youth and caregiver) and modeling approaches (second-order vs. bifactor). Our findings indicate that PS is related to general psychopathology symptoms. This research points to processing speed as an important transdiagnostic construct that warrants further exploration across development.

## Introduction

Comorbidity of mental health symptoms in youth is pervasive (Merikangas et al., 2009; Willcutt, 2014) and has led to interest in transdiagnostic risk factors for mental health symptoms. One way to represent general risk for psychopathology is with a latent construct, termed the *p* factor, which captures the common variance across a wide range of mental health symptoms (Caspi et al., 2014; Lahey et al., 2012). Cognition is a frequently examined correlate of the *p* factor. Both general cognition (IQ) and executive functions (EF) have shown a negative relationship with the *p* factor (Caspi et al., 2014). A related, but dissociable, cognitive construct, processing speed (PS), has received less research attention, despite PS’s link to a range of mental health symptoms (Willcutt et al., 2008). The present study addresses this gap by examining the association between general psychopathology (*p* factor) and a latent PS factor in a sample of youth. In what follows, we will (1) discuss existing literature on the *p* factor and cognition (EF, IQ), (2) define PS, its measurement, and relation to EF and IQ, and (3) discuss existing research on the relation between PS and psychopathology.

### *p* Factor Overview

High prevalence of comorbidity suggests that there may be a cohesive structure underlying psychological symptoms that are often considered distinct (Caspi & Moffitt, 2018). One innovation in conceptualizing comorbidity is an integrative factor of psychopathology that models systematic patterns of symptom co-occurrence. Theoretical perspectives on this general factor of psychopathology encompass two main lines of research: (1) a *p* factor model that typically includes internalizing and externalizing symptoms and may include other symptoms domains (e.g., thought disorders) (Caspi et al., 2014; Lahey et al., 2012) and (2) a Hierarchical Taxonomy of Psychopathology [HiTOP] model, which is a multi-layered model including a wider array of domains beyond mental health, such as personality, social skills, and physical health symptoms (Kotov et al., 2021). What is clear from the literature is that the general psychopathology factor emerges across these two lines of work which vary in measurement and statistical techniques (Caspi & Moffitt, 2018; Castellanos-Ryan et al., 2016). The current study is primarily interested in whether PS is significantly associated with a general psychopathology factor defined by the most common forms of psychopathology in youth, internalizing and externalizing symptoms, so a *p* factor model was most appropriate for this study.

### The *p* Factor and Cognition

There are many theoretical and empirical correlates of the *p* factor, including cognition, personality, emotion regulation, stress, thought problems, and sleep (Caspi & Moffitt, 2018; Castellanos-Ryan et al., 2016; Patalay et al., 2015). Cognition is one of the leading candidates due to both cross-sectional and prospective associations between cognition and various mental health symptoms (Beauchamp et al., 2022). Thus far, the *p* factor-cognition literature has largely focused on the neuropsychological constructs of EF and IQ. EF has been measured using tasks that assess working memory (WM), planning, organizing, attention, inhibition, and cognitive control, with resulting correlations in the small to moderate range with the *p* factor (*r* = −0.10– −0.29; Blanken et al., 2017; Huang-Pollock et al., 2017; Castellanos-Ryan et al., 2016). The range is similar for correlations found between the *p* factor and general cognition (*r* = −0.19– −0.34) (Caspi et al., 2014; Grotzinger et al., 2019; Harden et al., 2020). While these studies show reliable, albeit modest, correlations between EF/IQ and the *p* factor, the related cognitive construct of PS has received less research attention thus far.

### Processing Speed

PS can be thought of as a general mental efficiency that influences performance on a range of speeded tasks (Kail & Salthouse, 1994; Nigg et al., 2017). PS is a promising candidate as a cognitive correlate of the *p* factor because it shows robust associations with a wide range of mental health and neurodevelopmental disorders (Beauchamp et al., 2022). However, PS can be difficult to operationally define and measure because performance on a PS task depends on an individual’s skill with the task-specific cognitive demands and the speed at which one can execute those demands. In what follows we describe our approach to PS measurement, including our modeling strategy for isolating speed from other cognitive demands.

This study draws on the cognitive psychometric literature for PS conceptualization and measurement. In this literature, PS is defined as having at least two related subdomains: (1) cognitive speed (*Gs*) and (2) decision/reaction time (*Gt*) (McGrew, 2009; McGrew & Evans, 2004). Cognitive speed has been defined as “the speed with which children and adolescents execute basic cognitive processes” (Kail & Ferrer, 2007, p. 1760). Decision time refers to the speed of reacting to and making decisions in response to simple stimuli (e.g., simple reaction time, choice reaction time, inspection time) (McGrew, 2009; McGrew & Evans, 2004). Cognitive speed measures have been shown to be more strongly associated with learning and attention weaknesses in children, as compared to decision time measures (Jacobson et al., 2011; Naples et al., 2012). Cognitive speed measures are also commonly included in neuropsychological research and practice, such as the Wechsler intelligence scales (Coding, Symbol Search) and other standardized cognitive assessments (e.g., rapid naming, verbal fluency, visual search, and sequencing tasks), making cognitive speed measures most applicable to clinical practice. Because cognitive speed is more strongly implicated in youth psychopathology and is measured more commonly in clinical practice, this study focused on cognitive speed measures as opposed to decision time measures.

We used four cognitive speed measures (*Gs*; McGrew & Evans, 2004) in this study, two commonly used in neuropsychological and psychoeducational assessments (Wechsler Coding, Symbol Search) and two experimental measures (Colorado Perceptual Speed, Identical Pictures Test). All four tasks are paper-and-pencil, two-dimensional, visual tasks which require multiple cognitive operations and have written output, but they vary in their emphasis on other cognitive functions (e.g., graphomotor dexterity, automaticity of letter knowledge, short-term memory). As mentioned above, performance on cognitive speed tasks involves both skill with the required cognitive operation and the speed of completing that operation. As such, skill with the cognitive operation is usually a confounding factor for speed measurement. One way to isolate the effect of speed from other task demands is using latent factors which isolate the shared characteristics of tasks from the differing cognitive demands of tasks. Based on our task analysis, we conclude that our latent factor reflects the ability to quickly solve pattern recognition tasks and indicate the response in writing. The writing aspects of the latent factor are minimal, however, because three of the four tasks (Coding is the exception) have minimal written demands (putting a line through or circling a matching item), so the latent factor includes some aspect of fast written responses, but not the graphomotor dexterity that would be required for writing symbols. In that follows, when describing our study, we refer to this latent factor indexing aspects of cognitive speed as PS for simplicity and to align with the neuropsychological literature (i.e., processing speed index on the WISC-5 measured by Coding and Symbol Search).

While we have chosen the psychometric PS measurement tradition for this study, there are also complementary approaches in the cognitive science literature. Cognitive science often operationalizes speed by measuring reaction times to stimuli or efficiency of evidence accumulation (EEA), which is the rate at which an individual gathers relevant evidence in the environment to make an accurate decision (Weigard & Huang-Pollock, 2017). Though the two fields have different traditions in measuring speed, they both converge on PS being a critical cognitive function related to various other cognitive domains and mental health symptoms (Beauchamp et al., 2022; Weigard & Sripada, 2021).

### Processing Speed, Executive Functioning, and General Cognition

Cognitive speed is moderately correlated (around 0.5) with both IQ (Wechsler, 2014) and EF (Cepeda et al., 2013) indicating both overlap and distinctiveness of the constructs. A cognitive speed index is included in current Wechsler IQ measures (Processing Speed Index), but it has the weakest relation to Full Scale IQ of all the indices (*β* = 0.51 for PS with FSIQ vs. *β* = 0.81–1.0 for the four other indices with FSIQ) (Wechsler, 2014). The extent of the overlap of cognitive speed and EF is an open question in the literature (Cepeda et al., 2013), and largely dependent on measurement of both constructs. For example, many cognitive speed tasks likely also capture EF skills (e.g., working memory) and vice versa (e.g., some so-called EF tasks are speeded). As discussed above, latent models can also help with differentiating PS and EF. For this study, the latent factor primarily emphasizes cognitive speed because the speed demands are most salient across all tasks. However, there are also dimensions of EF that are required across all the tasks and thus could be present in the latent variable. For example, all four tasks require attention, though they do not require sustained attention given the short length of each task. All four tasks also require a small amount of decision-making. We would not expect other aspects of EF, such as inhibition, working memory, or shifting (Miyake & Friedman, 2012), to be strongly represented in our latent variable because those skills are not emphasized across all four tasks. Though attention and decision-making are expected to influence performance on the cognitive speed tasks, we also note that these demands are comparable to any cognitive task generally and not specific to the processing speed construct. In short, though components of EF may be reflected in the PS latent factor, we expect that the latent factor will primarily reflect cognitive speed given the most salient task demands.

The developmental trajectory of PS, EF, and IQ throughout childhood and adolescence can also provide insight on the relationships between constructs. For example, cross-sectional and longitudinal studies have established a developmental cascade of cognitive skills, where developmental increases in cognitive speed precede and drive performance on fluid reasoning and working memory (WM) measures (Fry & Hale, 1996; Kail, 2007; Kail et al., 2016; Weigard & Sripada, 2021). Additionally, a cognitive science study experimentally manipulated processing speed within subjects and found that speed impacted working memory performance, providing evidence for a directional relationship with speed driving WM (Weigard & Huang-Pollock, 2017). Together, these literatures underscore the potential developmental primacy of PS, where PS forms the foundation for further gains in reasoning and WM skills. As such, we can expect that PS will be related to IQ and EF, but the potential developmental primacy of PS motivates inclusion of this construct in further work examining how cognition relates to psychopathology symptoms. Importantly, previous studies discussed above reporting moderate correlations between the *p* factor and EF or IQ did not also include latent measures of PS, so it is not known whether these relationships might be partially or wholly attributable to PS. In line with the developmental primacy model, PS might account for the relationship between EF/IQ and the *p* factor, but it is also possible that PS may be a distinct additional correlate of the *p* factor above and beyond EF and IQ. In this study, we will specifically be able to test these hypotheses regarding the overlap of PS and IQ in association with the *p* factor.

### Processing Speed and Mental Health Symptoms

Across both neuropsychological and cognitive science literatures, studies have found that PS relates to various psychopathologies. For example, both cognitive speed and EEA are associated with a range of mental health symptoms and neurodevelopmental disorders, including ADHD, schizophrenia, depression, and behavioral difficulties (Weigard & Sripada, 2021; Willcutt et al., 2008). Most previous studies examined the association of PS with individual disorders, without accounting for general psychopathology. Thus, an open question that this study aims to address is whether PS is associated with general psychopathology, distinct disorders, or both.

Few previous studies specifically tested a relationship between the *p* factor and PS. Caspi et al. (2014) used cognitive speed measures in adults (WAIS-IV PS composite), and Bloemen et al. (2018) used psychomotor speed in youth (button clicking). Both found modest correlations with the *p* factor (*r* = −0.18 and −0.21 respectively), but neither used latent factors to isolate speed from other task-specific demands. Nigg et al. (2017) examined a latent factor of reaction times across EF tasks in adults and found a significant association with the *p* factor (*r* = −0.25). These initial studies support a modest correlation between the *p* factor and PS across a span of PS tasks.

### The Current Study

The current study is the first to examine the relationship between a latent PS factor and the *p* factor in youth. We used latent modeling in a large sample of youth with multiple measures and raters of psychopathology symptoms. Our primary hypothesis was that PS would be significantly, negatively associated with the *p* factor (i.e., slower PS associated with greater general mental health symptomatology). Though the primary focus was assessing the relationship between PS and the *p* factor, we conducted secondary, exploratory analyses to assess if PS is related to individual symptom domains after accounting for the *p* factor. We consider these exploratory due to important statistical caveats to consider in these models (Forbes et al., 2021; Lahey et al., 2021), discussed in the bifactor portion of the Results section. Finally, given the theory that PS may drive later cognitive development, we also conducted exploratory analyses to test if PS can account for the relationship between the *p* factor and general cognition.

## Method

### Participants

Participants were recruited as part of the Colorado Learning Disabilities Research Center (CLDRC), which is a community-based twin study with enriched recruiting for children with attentional and reading difficulties (DeFries, 1997; Willcutt et al., 2019). The sample for this study includes 795 participants, ages 11–16 (Table 1). The study design and recruitment methods have been documented previously (DeFries, 1997; Willcutt et al., 2019). In brief, twins living within 150 miles of metropolitan Denver were identified through 22 local school districts or through the state’s twin registry. For both recruitment sources, all twins were invited to participate with subsequent screening for eligibility. Caregivers completed a phone questionnaire to screen for history of reading or attention difficulties. Screening questions in the phone questionnaire included whether the child had experienced difficulty with learning or reading, had difficulties paying attention, a history of hyperactivity, or had ever been diagnosed with dyslexia or ADHD. Caregivers also completed a rating scale measure of DSM-IV symptoms of ADHD (Barkley & Murphy, 1998). In addition, parallel questionnaires were sent to each twin’s primary classroom teacher, with permission from the caregiver. If either member of a twin pair was determined to have a history of reading or attention difficulties, the pair was invited to participate in the study. A comparison group of twins in which neither twin met screening criteria for reading or attentional difficulties was also recruited. Twins in the comparison group were matched to twins with reading or attentional difficulties on age, zygosity, and sex as reported by the caregiver. Inclusion criteria were: (1) primarily English-speaking home, (2) no evidence of neurological problems or history of traumatic brain injury, (3) no known genetic disorders or syndromes, (4) no uncorrected visual impairment, and (5) not deaf or hard-of-hearing. The source dataset used for this manuscript originally included N = 1,002 participants. Additional inclusion criteria specific to this study included: (1) a Verbal IQ or Nonverbal IQ above 85 on the WISC-R or WISC-III (Wechsler Intelligence Scale for Children, Revised or 3^rd^ Edition) and a Full-scale IQ (FSIQ) above 70 (N = 55 exclusions); and (2) the participant had at least 50% of the psychopathology measurements completed to minimize missing data (N = 152 exclusions). The final sample consists of 795 children and adolescents (Table 1). Given that this is a twin sample, we used robust modeling techniques to adjust for family relatedness.

### Procedures

The Institutional Review Boards at the University of Colorado, Boulder (CU Boulder) and the University of Denver (DU) approved the ongoing study protocol. Participants completed two testing sessions, the first at CU Boulder and the second at DU approximately two months later (median days between testing = 66 days). The testing session at CU Boulder included all PS measures used in this study, except for Symbol Search which was collected at DU. Child self-report psychopathology symptoms were collected at CU Boulder. Caregiver-report of child psychopathology occurred at DU for interviews and between the two visits for questionnaires. Testing was conducted by trained examiners who were research assistants with bachelor’s degrees or doctoral level clinical psychology graduate students. Examiners were trained to be sensitive to fatigue and offer small breaks and behavioral support to maintain motivation. Children taking stimulant medication were asked to discontinue use 24 h before testing unless instructed otherwise by their physician.

### Mixed Reporter Approach to the *p* Factor

There are multiple ways to model the *p* factor given the multiple measures and raters in this sample (see Table 2). A novel modeling contribution of this study was the use of a mixed-reporter approach. Most *p* factor models to date are single-reporter models. Studies that use more than one reporter typically either combine reports (e.g., model caregiver-report and child-report of anxiety on the same specific factor) (Laceulle et al., 2015) or take the higher symptom score of the two reporters (Lahey et al., 2012). Given that there is generally low to moderate agreement on childhood psychopathology symptoms across raters (De Los Reyes et al., 2015), our primary *p* factor model used a mixed-reported approach informed by clinical best practice and scientific evidence where youth reported on internalizing symptoms (Kemper et al., 2003) and caregivers reported on externalizing symptoms (Smith, 2007).

Our a priori choice to use this mixed-reporter model was supported by analyses that indicated better convergence between measures of internalizing symptoms for youth-report (mean correlation = 0.53, range = 0.40–0.67) compared to caregiver-report (mean correlation = 0.34, range = 0.12–0.68). For externalizing symptoms, we obtained caregiver-reports from two caregivers when possible. Though we had initially planned to include both reporters on externalizing scales in our models, models that included both caregivers’ ratings did not converge and had poor model fit (Table S2). Therefore, we used the caregiver report (> 98% of whom identified as women) with the most complete data (< 2% missing compared to 21–32% missing for the other caregiver). Correlations among caregiver-reported externalizing measures were moderate (mean correlation = 0.55, range = 0.39–0.76), consistent with previous literature (De Los Reyes et al., 2015). Given the novelty of using two reporters (youth-report and caregiver-report) for different domains in the same *p* factor model, we also ran secondary analyses using only caregiver-report for all symptoms to compare to the literature.

### Measures

All measures used in this study were modeled as dimensional symptoms (Clark et al., 2017).

#### Internalizing Measures

Child internalizing psychopathology was assessed using child self-report measures (Table 2).

#### Externalizing and Attention Measures

Child externalizing psychopathology was assessed using caregiver-report measures (Table 2). Note that the DICA Conduct module and the CBCL delinquency subset of questions (*r* = 0.75) have several items that are nearly identically worded (6/14 of the DICA items, 7/13 of the CBCL items). These two scales were thus combined; DICA CD questions were set to a scale of 0/2 for yes/no answers to match the CBCL scale of 0–2.

We tested two alternative models of externalizing symptoms, one that modeled the domain as one factor and one that separated externalizing and attention/hyperactivity into two factors. Attention/hyperactivity symptoms are frequently grouped with externalizing symptoms in the existing literature, but some evidence indicates better model fit when attention is a separate factor (Brikell et al., 2020), and previous factor analyses show lower loadings of attention than other behavioral symptoms on the externalizing domain (Achenbach & Rescorla, 2001; Greenbaum & Dedrick, 1998). We refer to the attention/hyperactivity factor as the “attention domain” for brevity.

#### Processing Speed

A processing speed factor was created using the following PS measures: WISC-III/R Coding, WISC-III Symbol Search, Colorado Perceptual Speed Test, and the Identical Pictures Task. Raw scores were used for all tasks. Older versions of the WISC PS tasks were administered because the study has been running for several decades and has prioritized consistency in measures over time to maximize sample sizes. WISC Coding requires the participant to rapidly copy symbols associated with numbers based on a key (test–retest reliability = 0.72, Wechsler, 1974, 1991). For this task, 39% of participants received the WISC-III and 61% received the WISC-R. Though participants completed different versions of the WISC Coding task, raw scores were appropriate to use in this case because the key and the time limit for each WISC Coding version is the same. WISC Symbol Search requires the participant to rapidly mark a target symbol based on presence of a matched symbol (test–retest reliability = 0.81, Wechsler, 1991). 100% of participants received the WISC-III version for this task. The Colorado Perceptual Speed Test requires participants to quickly identify a target string of letters or letters and numbers among three foils (test–retest reliability = 0.81, Decker, 1989; DeFries et al., 1981); the two subtests with non-pronounceable letter strings were used to minimize the effect of individual differences in reading skill. The Identical Pictures Test requires the participant to quickly identify a target picture among an array of pictures with four foils (test–retest reliability = 0.82, French et al., 1963). All four measures of PS are moderately correlated (*r* = 0.41–0.56) after accounting for age, age-squared, and caregiver-identified sex, and have been used in previous research as a PS factor (McGrath et al., 2011; Peterson et al., 2017).

#### General Cognition

In secondary analyses, we included a measure of general cognition as a covariate. We created a latent factor of eight WISC-III (39%) or WISC-R (61%) subtests to capture general cognition, including scaled scores from four nonverbal reasoning tasks (block design, object assembly, picture completion, picture arrangement) and four verbal comprehension tasks (vocabulary, similarities, comprehension, and information). Note that this general cognition construct is an overly conservative covariate with respect to PS because all four nonverbal reasoning tasks have a speeded component.

### Data Cleaning and Analysis

Raw scores were used for PS and psychopathology measures (Table S1), residualizing for age, age-squared, and caregiver-identified sex. Age-squared was included as psychopathology and PS development can have nonlinear features. Histograms were inspected for normality and outliers were winsorized to four standard deviations. While some psychopathology variables were originally symptom counts, histograms of almost all scales approached normality (skew and kurtosis < 3) after residualizing; as such, we proceeded with analyses for continuous data rather than count data. One scale (conduct/delinquency symptoms) had kurtosis > 3 and was transformed using the square root transformation. Before analyses, we screened for missing data. We set an item criterion that a scale had to have > 80% of items answered or else that scale was set to missing. For all measures, scale-level missing data was minimal (< 2% missing), apart from youth self-report (YSR) (12% missing) and Symbol Search (24%). Symbol Search had the highest missingness because it was added to the battery later.

Confirmatory factor analyses (CFA) were run with Mplus version 8.4 using maximum likelihood estimation with robust standard errors (MLR) and missing data was handled with full information maximum likelihood estimation. The familial relationships in our sample violates statistical assumptions of independence. This dependency be successfully modeled using MLR estimation with clustering correction in the ‘Complex’ option in Mplus (Rebollo et al., 2006). Model fit for all CFA models were assessed using the following guidelines: Comparative Fit Index (CFI) > 0.90, Root Mean Square Error of Approximation (RMSEA) < 0.08, and Standardized Root Mean Square Residual (SRMR) < 0.08 (Hu & Bentler, 1999; Browne & Cudeck, 1992; Cangur & Ercan, 2015). Nested models were compared using the Satorra-Bentler scaled chi-square difference test (Satorra & Bentler, 2010). For non-nested models, Bayesian Information Criterion (BIC) and Akaike’s Information Criterion (AIC) were used as indices of absolute fit.

### Model‑Building & Alternative Models

Previous research on the *p* factor has typically used a bifactor model. However, recent statistical concerns (e.g., inflated fit statistics, anomalous results) with the bifactor model (Eid et al., 2017; Mansolf & Reise, 2017) led us to choose a second-order model as our primary model. Second-order models are related to but distinct from bifactor models and do not have the same statistical concerns. Briefly, second-order models capture covariation between first-order factors (e.g., internalizing, externalizing, and attention), while bifactor models capture covariation between the indicators themselves (e.g., CBCL Aggression, CDI Total etc.) (Mansolf & Reise, 2017). The biggest conceptual difference between the two models lies in the meaning of the subdomains, which are not the main focus in this study. We also considered an S-1 bifactor model but decided this modeling approach was not conceptually appropriate for this study given the need to designate what the *p* factor represents a priori (Eid, 2020; Heinrich et al., 2021).

To create the primary second-order, mixed-reporter *p* factor model, we followed several model-building steps to determine the substructure, including a one-factor, two-factor (internalizing/externalizing), three-factor (internalizing/externalizing/attention), and second-order model. To facilitate direct comparisons to previously published studies, we explored three alternative secondary models, (1) mixed-reporter, bifactor; (2) caregiver-only report, second-order; and (3) caregiver-only report, bifactor.

## Results

### First‑Order Domains and the *p* factor

We compared one-, two-, and three-factor correlated traits models to determine the substructure of the *p* factor. The model with three factors (internalizing, externalizing, attention) was the superior model based on the Satorra-Bentler scaled chi-square difference test (Table S3). All three first-order factors exhibited strong reliability, measured by the omega subscale (ω_s_) values (0.85, 0.84, and 0.86 for internalizing, externalizing, and attention domains respectively; ideally ω_s_ > 0.75) (Forbes et al., 2021; McDonald, 1999). Correlations between the first-order factors were *r*(41) = 0.25 for internalizing and attention, *r*(41) = 0.21 for internalizing and externalizing, and *r*(41) = 0.72 for externalizing and attention, *p* < 0.001 for all three relationships. During model building, two modification indices (MI) were indicated and rejected for theoretical reasons. In the two-factor model, MIs indicated a residual correlation between two attention scales (CBCL Attention Problems and DBRS Inattention), which was consistent with our a priori plan to test a three-factor model with attention as a separate domain. In the three-factor model, MIs suggested cross-loading hyperactivity/impulsivity symptoms onto the externalizing factor. While this cross-loading has been included in some previous research (Harden et al., 2020), we opted to keep hyperactivity as an indicator of the attention factor because this was the higher loading.

Once the substructure of the *p* factor was established, we loaded these three factors (internalizing, externalizing, attention) onto a second-order general psychopathology factor for the primary model (Figure S1). We also constructed three secondary, alternative models of the *p* factor (mixed reporter, bifactor; caregiver-only, second-order; caregiver-only, bifactor) to assess generalizability of results and facilitate comparisons with existing literature (Tables S4–7). The primary model and three secondary models are generally comparable, with the exception of internalizing loadings. For both second-order and bifactor models, internalizing loadings onto the *p* factor are lower for mixed-report vs. caregiver-only report models (e.g., second-order model: *β* = 0.27 vs. *β* = 0.81 respectively). Finally, reliability estimates (Table S8) for bifactor models indicated excellent reliability for the total model, acceptable reliability for the *p* factor, and generally less reliable specific factors, consistent with previous literature (Watts et al., 2019).

### *p* Factor and Processing Speed

**Fig. 1:**
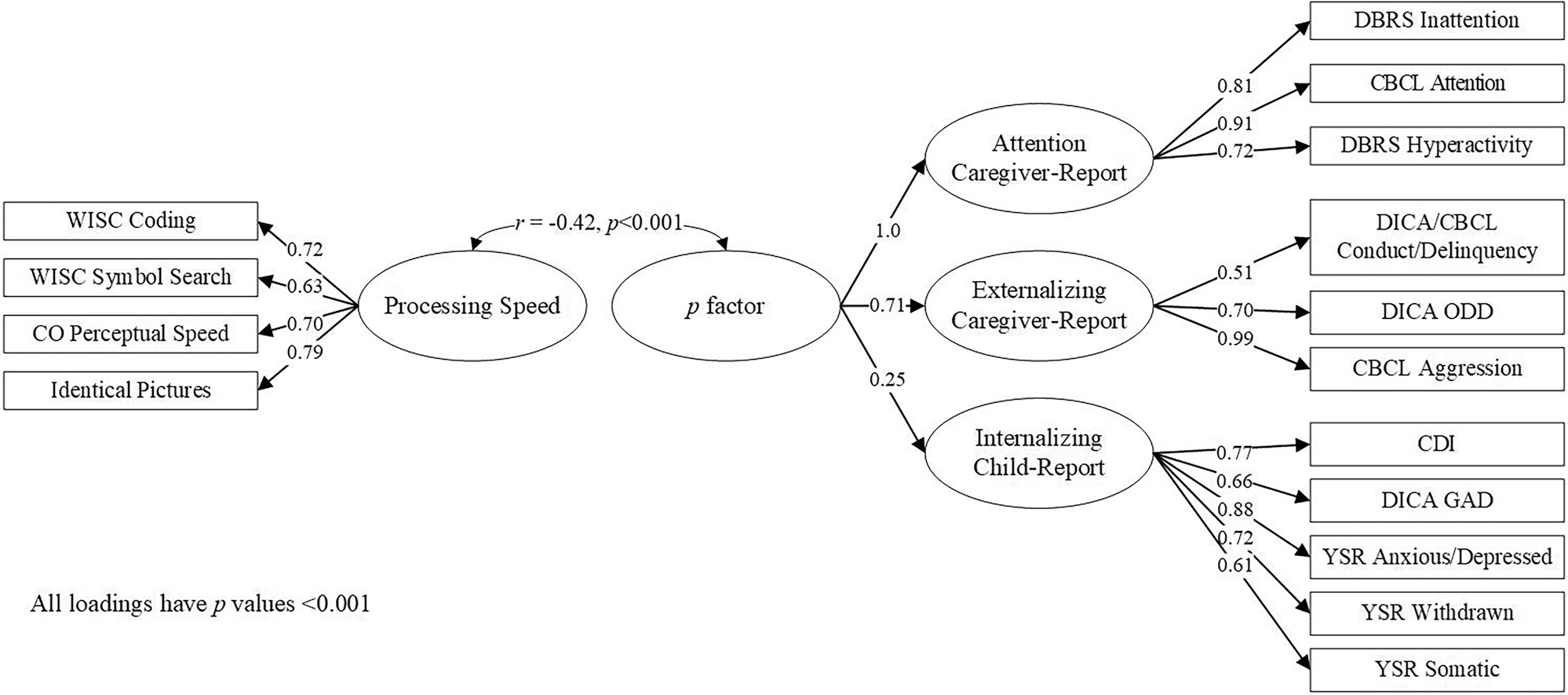
Standardized Loadings for the Second-Order, Mixed-Reporter *p* factor and Correlation with Processing Speed

The one-factor CFA for the four PS measures had excellent model fit (Χ^2^(2) = 0.99, *p* < 0.001, CFI = 1.0, RMSEA = 0.00 [90% CI 0.00–0.06], SRMR = 0.01). We then correlated the PS factor with the three subdomain factors in a correlated traits model. The PS factor was significantly associated (*p* < 0.01) with the three factors (*r*(84) = −0.14 with internalizing, −0.27 with externalizing, and −0.43 with attention; Figure S2). We then correlated the PS factor with the *p* factor from the primary second-order model. This model would not converge because the standardized attention loading on the *p* factor exceeded 1 (*β* = 1.019), which was not surprising given the high loading (0.93) in the second-order *p* factor model (Figure S1). We fixed the standardized loading to 1 given that the negative residual variance of the attention factor was not statistically greater than 0. This model fit the data well (Χ^2^(87) = 249, *p* < 0.001, CFI = 0.96, RMSEA = 0.05 [90% CI 0.04–0.06], SRMR = 0.04). PS was significantly correlated with the *p* factor (*r*(87) = −0.42, *p* < 0.001) (Fig. 1).

### Alternative Models of the *p* Factor

After completing the primary analyses, we then examined the correlation between PS and the *p* factor in the three secondary models: (1) mixed-reporter, bifactor; (2) caregiver-only report, second-order; (3) caregiver-only report, bifactor. PS was significantly correlated with the *p* factor in all models, showing remarkably stable results across raters and modeling strategies (Table 3).

Using our mixed-reporter, bifactor model, we conducted exploratory analyses to assess if PS is related to domain-specific variance in psychopathology factors after accounting for the *p* factor. We used the bifactor model instead of the second-order model because bifactor models have a strength in defining domain-specific variance (Lahey et al., 2021). We examined correlations between PS and the internalizing and externalizing specific factors after accounting for the *p* factor (note that attention did not have residual variance as described above). PS was *not* significantly related to the domain-specific factors after accounting for the *p* factor (*r*(81) = −0.03, *p* = 0.48 for internalizing, *r*(81) = 0.06, *p* = 0.14 for externalizing) (Table S6). These analyses should be considered exploratory because the residual variance of specific factors in bifactor models can be statistically unreliable and theoretically difficult to define. In our case, the reliability of the internalizing specific factor was acceptable (ω_hs_ = 0.79), but the reliability of the externalizing specific factor does not meet current statistical thresholds for acceptability (ω_hs_ = 0.40) (see Table S8). As such, we present these analyses for comparison and replication, and we caution against over-interpretation (Forbes et al., 2021).

### Accounting for Covariates

Given the high loading of attention measures onto the *p* factor and the known link between slower processing speed and greater ADHD symptomatology (Kramer et al., 2020; McGrath et al., 2011), we conducted a secondary analysis to assess whether the correlation between PS and the *p* factor was primarily due to ADHD measures that were included in the *p* factor model. We created a mixed-reporter, second-order *p* factor model with just internalizing and externalizing measures, leaving out measures of attention/hyperactivity (Table S9). This model fit the data well (Χ^2^(51) = 104, *p* < 0.001, CFI = 0.98, RMSEA = 0.04 [90% CI 0.03–0.05], SRMR = 0.03), and the strength of the association between PS and the *p* factor (*r*(51) = −0.42, *p* < 0.001) was the same as in the primary model.

Additionally, we ran secondary analyses controlling for general cognition (Table S10), which is related to both PS and the *p* factor (Beauchamp et al., 2022; Grotzinger et al., 2019). Our general cognition factor was correlated with the *p* factor (*r*(225) = −0.24, *p* < 0.001) and with PS (*r*(225) = 0.62, *p* < 0.001). The correlation between PS and the *p* factor (*r*(225) = −0.42) was significantly stronger than the correlation between general cognition and the *p* factor (*r*(225) = −0.24), based on Fisher’s z-statistic test (z = 4.04, p < 0.001) (Dunn & Clark, 1969). When general cognition and PS were both included in a model predicting the *p* factor, PS continued to be significantly associated with the *p* factor (*β* = −0.44, *p* < 0.001), while general cognition was no longer significantly associated with the *p* factor (*β* = 0.03, *p* = 0.6). These results indicate that the relationship between general cognition and the *p* factor may be due to their mutual relationship with PS.

Finally, we ran a secondary analysis using age and sex as covariates of latent PS and psychopathology to understand the influence of these variables on the relationship between psychopathology and PS (Table S11). Previous models, described above, had accounted for age and sex prior to model-building. For this analysis, we constructed the *p* factor model and the PS factor using raw scores uncorrected for age and sex. We then used age and sex as predictors of the latent psychopathology and PS factors. Consistent with previous PS literature (Camarata & Woodcock, 2006), we found that older youth and females were significantly faster on average. Somewhat surprisingly, age was not related to the *p* factor, nor was it related to any of the included first-order domains (internalizing, externalizing, or attention factors) in the correlated traits model, possibly because of the restricted age range in this study (11–16 years). Sex was significantly related (small effect size) to the *p* factor, externalizing symptoms, and attention, with males having higher symptoms (Table S11). Sex was not related to the internalizing factor. Most importantly, the correlation between PS and psychopathology was similar (*r(*113) = −0.44, *p* < 0.001) with this alternative model-building approach where age and sex were explicitly modeled, providing good convergence with the primary result.

## Discussion

This study is the first to test whether a latent processing speed factor is related to the *p* factor in youth. Results showed that the PS factor was significantly, negatively associated with the *p* factor, indicating that slower PS is associated with increased general psychopathology in youth. We found similar correlations (*r* ~ −0.42) between the *p* factor and PS across various modeling approaches encompassing different raters (mixed-reporter, caregiver only) and hierarchical models (second-order, bifactor), which speaks to the stability and generalizability of the correlation.

### Processing Speed and Psychopathology

The significant correlation between the *p* factor and PS expands the breadth of mental health symptoms that should be explored in relation to PS. PS is most frequently included in studies examining neurodevelopmental disorders (McGrath et al., 2011; Peterson et al., 2017), but this study, along with existing work, suggests that PS might have broader relationships with internalizing and externalizing disorders (Willcutt et al., 2008; Nigg et al., 2017; but see Calhoun & Mayes, 2005 for a different view). In the existing work showing associations between PS and specific mental health disorders, studies do not usually account for general mental health, making it difficult to know whether associations occur because PS is related to mental health generally, specific disorders, or both. The present study adds to the existing literature by suggesting that PS is related to mental health generally, providing some support that PS could be a pervasive correlate related to a wide range of mental health symptoms. These results suggest that PS may be a transdiagnostic mechanism with implications for prevention and early intervention for general psychopathology symptoms. Unfortunately, the cross-sectional nature of this dataset limits conclusions about causal directionality between PS and mental health. Future research should include longitudinal work to assess the developmental unfolding of this relationship. Furthermore, this study cannot speak to potential neurobiological mechanisms behind the PS/p-factor relationship, but one plausible hypothesis is that PS and various psychopathologies are related because they are both related to white matter connectivity (Nigg et al., 2017; Thomason & Thompson, 2011). Future research across multiple levels of analysis (neurobiological, cognitive, and behavioral) is needed to develop a fuller understanding of the PS/*p*-factor relationship.

Secondary, exploratory analyses examined the relationship between PS and specific psychopathology domains after accounting for the *p* factor (i.e., bifactor model). These domain-specific relationships were not significant, suggesting that the association between PS and psychopathology is strongest for the general factor. We caution against over-interpretation of this result given the lack of reliability and stability of specific factors after extracting general variance (Eid, 2020; Forbes et al., 2021). Due to the statistical limitations of bifactor models, we cannot fully rule out domain-specific associations between PS and specific psychopathology.

### *p* Factor and Cognition

PS, EF, and IQ are overlapping, yet distinguishable cognitive constructs; thus, disentangling their general and specific relationships with mental health symptoms is important. The correlation between PS and the *p* factor (*r* = −0.42) was stronger than previously reported correlations of the *p* factor with EF and IQ (*r* ~ 0.1–0.3) (Caspi et al., 2014; Grotzinger et al., 2019), indicating that PS might account for more variance in general mental health than other aspects of cognition. Indeed, this pattern was found in our sample, as the correlation between the *p* factor and our latent general cognition factor (*r* = −0.24) was weaker than with our latent PS factor (*r* = −0.42). When both PS and general cognition were included in the same model as predictors of the *p* factor, PS contributed uniquely above and beyond general cognition, but general cognition did not contribute uniquely above and beyond PS. This analysis indicates that the relationship between general cognition and mental health previously found in the *p* factor literature may be attributable to PS, aligning with both theoretical and empirical literature positing PS as a developmental driver of general cognitive skills, especially fluid reasoning (Fry & Hale, 1996; Kail, 2007). This study did not directly examine EF, so future research should examine all three cognitive constructs (PS, IQ, EF) to determine general and specific associations with the *p* factor.

### Modeling Approach

Strengths of our modeling approach included (1) latent measurement of both PS and psychopathology, (2) using a second-order model in primary analyses with convergence from bifactor models, and (3) including multiple raters, with child-report of internalizing symptoms and caregiver-report of externalizing symptoms. It was important that we used a latent modeling strategy for PS given confounding cognitive skills that influence PS measurement. Further, while second-order models have been used in some previous *p* factor literature (Michelini et al., 2019), most studies used bifactor models which have received scrutiny for model instability and inflated fit statistics (Eid et al., 2017; Mansolf & Reise, 2017). Modeling methodology is evolving and each model (e.g., bifactor, second-order) has strengths and weaknesses (Eid, 2020; Heinrich et al., 2021). The fact that we found similar correlations between PS and the *p* factor using both second-order and bifactor models speaks to the robustness of the finding.

Our decision to use multiple raters was in line with best clinical practice and research (Kemper et al., 2003; Smith, 2007), with the child reporting on internalizing symptoms and the caregiver reporting on externalizing symptoms. Previous *p* factor literature has examined child-report or caregiver-report, but they have mostly been in separate models (for exceptions see Laceulle et al., 2015; Lahey et al., 2012). One common concern regarding the *p* factor is that it may be overly influenced by individual differences in reporting style, such as a positive or negative skew when reporting symptoms (termed halo effect) (Caspi & Moffitt, 2018). Using two reporters of different symptoms domains in one model can help address this concern. We encourage consideration of this second-order, mixed-reporter model in future *p* factor literature given the model strengths (e.g., raters aligning with best practice; removal of potential halo effect), as well as the convergence of results across models.

### Unexpected Modeling Results

Across our primary and secondary *p* factor models, we found a few unexpected results deserving further comment. We observed a difference in the loading of internalizing symptoms based on whether we used a single-reporter (*β* = 0.81) or mixed-reporter (*β* = 0.27) second-order model of the *p* factor. There are a few potential explanations for why the loading may be lower in the mixed-reporter model. First, this discrepancy may reflect a lack of convergence between youth and caregiver reports (De Los Reyes et al., 2015) coupled with the fact that internalizing symptoms were reported by the child, whereas both externalizing and attention were reported by the caregiver, potentially weighting the *p* factor toward the caregiver. Alternatively, this result might indicate a true difference in the relationships between internalizing and externalizing symptoms with the *p* factor. It is difficult to assess existing evidence for this hypothesis because there is a lack of convergence of internalizing loadings in previous *p* factor literature (lower loadings ranging from *β* = 0.13–0.46, higher loadings ranging from *β* = 0.72–0.90) (Castellanos-Ryan et al., 2016; Laceulle et al., 2015; Michelini et al., 2019). While we cannot resolve why the internalizing loading was lower in the mixed-reporter vs. single-reporter model, we report the loading discrepancy as an important consideration for future work, especially given the prevalent use of single-reporter models to date. For the purposes of our central question, we note that the correlation between PS and the *p* factor was stable across mixed-rater versus single-rater models.

Consistent with previous factor analyses of psychopathology (Achenbach & Rescorla, 2001), model fit was better when attention was a distinct first-order domain than when included with externalizing symptoms. One interesting result is that when looking across all four *p* factor models (second-order/bifactor; mixed-rater/single-rater), the loadings of attention measures on the *p* factor were very high, to the degree of suggesting that the *p* factor and attention may be synonymous constructs. This high loading, also found by Brikell et al. (2020), warrants further investigation. It is consistent with both theory and scientific evidence suggesting that attention is a key transdiagnostic correlate that is relevant to a range of psychopathology symptoms (Aitken & Andrade, 2021; King et al., 2013; Racer & Dishion, 2012). In response to the attention loading being so high, we completed a secondary analysis where we dropped the attention/hyperactivity measures from the *p* factor to ensure that the correlation between PS and the *p* factor was not solely due to these measures. The resulting correlation between PS and the modified *p* factor (*r* = −0.42, *p* < 0.001) was similar to the primary result, providing evidence that PS is associated with general mental health symptoms apart from the known association with attention/hyperactivity.

### Limitations and Future Directions

This study had several strengths, including a large sample size, multiple measures of psychopathology and PS, and use of latent modeling. However, our findings should be interpreted in the context of several limitations. First, our study is limited by the recruitment of participants in the study. This sample has less socioeconomic, racial, and ethnic diversity than the United States population, limiting the generalizability of the findings. Future research should include more diverse samples and consider the influence of self-reported gender. Additionally, the study does not include measures outside of internalizing, externalizing, and attention domains (e.g., psychosis, autism spectrum disorder, OCD). Further, we focus on cognitive speed tasks in this study, which represents one measurement tradition for PS. Because there are many different measurement approaches, we cannot be certain that these results would generalize to other forms of speed (e.g., EEA, decision time). However, there is extensive psychometric literature indicating that various types of speed load highly onto a general speed factor (McGrew & Evans, 2004; Salthouse, 1996), providing some evidence to expect generalization. Future research should examine the relationships between the *p* factor and other types of PS measurement.

In addition to measurement limitations, this dataset is enriched for attention and reading difficulties, both of which have been shown to be related to PS (McGrath et al., 2011). However, in practice, we have observed that it is a “soft selection” as those recruited for potential attention and reading challenges often do not meet criteria for a disorder, and those who were recruited in the control group often have undetected ADHD and reading difficulties, resulting in relatively normal distributions of these skills. In the current sample, 23% of children met symptom criteria (more liberal than full diagnostic criteria) for ADHD based on the OR rule from symptoms ratings (i.e., at least 6/9 ADHD symptoms from parent report OR teacher report [Lahey et al., 1994]). Given the slight enrichment for ADHD, a question is whether this sample has higher-than-expected rates of other psychopathology which could artificially strengthen the covariance of mental health symptoms and therefore the *p* factor. However, this does not seem to be the case, as clinically significant rates of symptoms are 9% for depressive symptoms (CDI ≥ 13 symptoms), 13% for internalizing symptoms broadly (child-report YSR T ≥ 70), and 4% for externalizing symptoms broadly (parent-report CBCL T ≥ 70). While some of these rates are higher than diagnostic rates in epidemiological studies (Merikangas et al., 2009), we would expect this as these are symptom counts and not diagnostic interviews. Thus, the elevations do not seem to indicate much higher than expected rates of psychopathology. Secondly, while our attention loadings are higher than some previous studies, we note that another *p* factor model in a population-based sample of youth showed similarly high loadings for attention symptoms (Brikell et al., 2020). The high loading for attention is also consistent with other theoretical (Racer & Dishion, 2012) and empirical work (King et al., 2013) showing that attention is a transdiagnostic correlate of internalizing and externalizing symptoms. Taken together, these studies provide some assurance that our *p* factor is consistent with previous literature and our high attention loading is not entirely due to the sampling.

The sample consists of twins who are at higher risk for preterm birth, which has been linked to lower PS, lower cognitive scores, and increased mental health challenges (Beauchamp et al., 2022). To understand whether this sampling approach biased our results, we compared our findings to those from non-twin samples. Our association between the *p* factor and general cognition (*r* = −0.24) mirrors previous findings in non-twin samples (*r* = −0.19– −0.34) (Caspi et al., 2014; Grotzinger et al., 2019), providing some reassurance that a higher incidence of preterm birth did not exert a strong upward bias on the correlation between PS and the *p* factor. Ultimately, however, it is important to replicate this finding in an independent, non-twin dataset.

Finally, there was a median of 66 days between testing sessions. At the first study visit, parents received child psychopathology questionnaires to complete at home and bring to their next study visit. Thus, it is possible that caregivers completed some ratings of child psychopathology up to two months, on average, before collection of youth-report measures. In practice, we found that many families filled out the questionnaires immediately before they needed to bring them to their next study visit, which was the visit at which children filled out their own psychopathology measures. However, to the extent that the measures could be separated in time, this would make finding a *p* factor more difficult and could attenuate the correlation with PS. We found a robust relationship between the *p* factor and PS, but we note that it could be stronger if data were consistently collected at the same timepoint.

In conclusion, this study was the first to examine the relationship between a latent PS factor and the *p* factor in a sample of youth. We found a significant, moderate association between PS and the *p* factor (*r* = −0.42) that was stable across different raters and different modeling techniques. This study expands the existing literature examining PS in relation to specific disorders by showing that PS is related to what is *shared* across psychopathology. The association with the *p* factor was stronger for PS than general cognition, both in this sample and when comparing to previous correlations with IQ, indicating that PS could be an especially important transdiagnostic construct that warrants further attention and investigation.

## Supplementary Material

Supp_1

Supp_2

Supp_3

## Figures and Tables

**Table 1:** Participant Demographics

*Participant Demographics*	Mean	SD	Range

Age	13.4	1.7	11.0–16.9
Mean caregiver years of education	15.8	2.6	10.0–28.0
Full Scale IQ (WISC-R/III)	106.9	13.3	70.0–144.0
***Sex as identified by caregiver*** ^a^	**N**		
Female	431		
Male	364		
***Race***	**Wave 1**	**Wave 2**	
	(1995–2006)^c^^,^^d^	(2006–2011)^c^^,^^d^	
Asian	< 2%	< 2%	
Black	< 2%	< 2%	
Hispanic or Latino/a/x	2.6%	–	
Multiple groups identified^b^	10.1%	9.3%	
Native American/American Indian/Alaska	< 2%	< 2%	
Native/Indigenous			
White	86.4%	86.8%	
Prefer to self-describe	< 2%	2.0%	
***Ethnicity*** Hispanic or Latino/a/x		3.1%	
Multiple ethnicities identified^b^	–	7.8%	
Not Hispanic or Latino/a/x	–	89.1%	

a We assessed sex as binary and did not include intersex as an option. We did not assess self-reported gender. We have revised our demographic data collection to be more inclusive of gender identities

b In earlier phases of data collection, caregivers self-reported race and ethnicity for themselves but not their children. Here we indicate if caregivers endorsed multiple identifications. We want to note the limitations of this approach, however, as it does not capture the identification that families and children would choose for the child. We have revised the race and ethnicity data collection to align with current best practices for inclusiveness in research studies (e.g., Wadsworth et al., 2016)

c Our earliest waves of data collection included a single variable that combined race and ethnicity, consistent with federal guidance at the time. Since 2006, we have been collecting race and ethnicity information separately

d For additional confidentiality protections for participants, if the percentage representation of a group is less than 2%, we indicate < 2%

**Table 2:** Indicators for Psychopathology Symptom Dimensions

Measure	Reported Test-retest Reliability	Cronbach’s alpha: current sample	Brief Description	Sample Item	Reference

***Child-report Internalizing***					
CDI	0.66–0.82	0.83	Self-report of depressive symptoms	Ex. I am sad all the times, many times, or once in a while	Kovacs (1981)
DICA GAD module	0.54	0.72	Self-report of generalized anxiety symptoms, worry across contexts	Ex. I worry a lot about doing things perfectly	Boyle et al. (1993)
YSR - anxious/depressed	0.74	0.83	Self-report ratings of anxiety and depression symptoms	Ex. I feel too guilty	Achenbach and Rescorla (2001)
YSR - withdrawn	0.67	0.64	Self-report ratings of withdrawn symptoms	Ex. I am too shy or timid	Achenbach and Rescorla (2001)
YSR - somatic	0.76	0.72	Self-report ratings of somatic symptoms	Ex. Nausea without known medical cause	Achenbach and Rescorla (2001)
***Caregiver-report Externalizing***					
DICA ODD module	0.67	0.77	Caregiver interview regarding symptoms of oppositional defiant disorder	Ex. Often argues with parents, teachers, or other adults	Boyle et al. (1993)
DICA CD module	0.87	0.66	Caregiver interview regarding symptoms of conduct disorder	Ex. Have they ever stolen anything without the person noticing	Boyle et al. (1993)
CBCL - aggressive behavior	0.90	0.90	Caregiver ratings of aggressive behaviors	Ex. Gets in many fights	Achenbach and Rescorla (2001)
CBCL - delinquency	0.91	0.78	Caregiver ratings rule-breaking behaviors	Ex. Lying/cheating	Achenbach and Rescorla (2001)
Combined scale: DICA CD/CBCL delinquency	N/A	0.78	Caregiver ratings of conduct and delinquency	Ex. Skips school	Created for this manuscript
***Caregiver-report Attention/Hyperactivity***					
DBRS Inattentive	0.71–0.94	0.94	Caregiver ratings of DSM-IV inattentive symptoms	Ex. Fails to give close attention or makes careless mistakes	Barkley and Murphy (1998)
DBRS Hyperactive/Impulsive	0.66–0.93	0.88	Caregiver ratings of DSM-IV hyperactive/impulsive symptoms	Ex. Often “on the go” or as if driven by a motor	Barkley and Murphy (1998)
CBCL - attention problems	0.92	0.84	Caregiver ratings of inattention	Ex. Fails to finish things they start	Achenbach and Rescorla (2001)

*CDI* Child Depression Inventory, *DICA GAD* Diagnostic Interview for Children and Adolescents, Generalized Anxiety Disorder module, *YSR* Youth Self Report, *ODD* Oppositional Defiant Disorder, *CD* Conduct Disorder, *CBCL* Child Behavior Checklist, *DBRS* Disruptive Behavior Rating Scale

**Table 3:** Correlation of Processing Speed and the *p* factor Across Four Models

	Second-Order Model	Bifactor Model

Mixed-reporter (child-report internalizing, caregiver-report externalizing and attention)	***r*(87) = −0.42, *p* < 0.001**	*r*(81) = −0.43, *p* < 0.001
Caregiver-report only for internalizing, externalizing, attention	*r*(86) = −0.40, *p* < 0.001	*r*(79) = −0.42, *p* < 0.001

* The primary model (main result) is bolded. The other three models are included to compare to the literature

## Data Availability

Cognitive and academic data from the CLDRC (Colorado Twin Project) are publicly available through LDbase, a learning and developmental data repository:https://www.ldbase.org/. Mplus code for the models is available upon request from the corresponding author.
