# Supplementary figures and images for: Processing Speed is Related to the General Psychopathology Factor in Youth
Source: Res Child Adolesc Psychopathol. Author manuscript; Available in PMC 2024 Aug 1. (PMC10368543; doi:10.1007/s10802-023-01049-w)

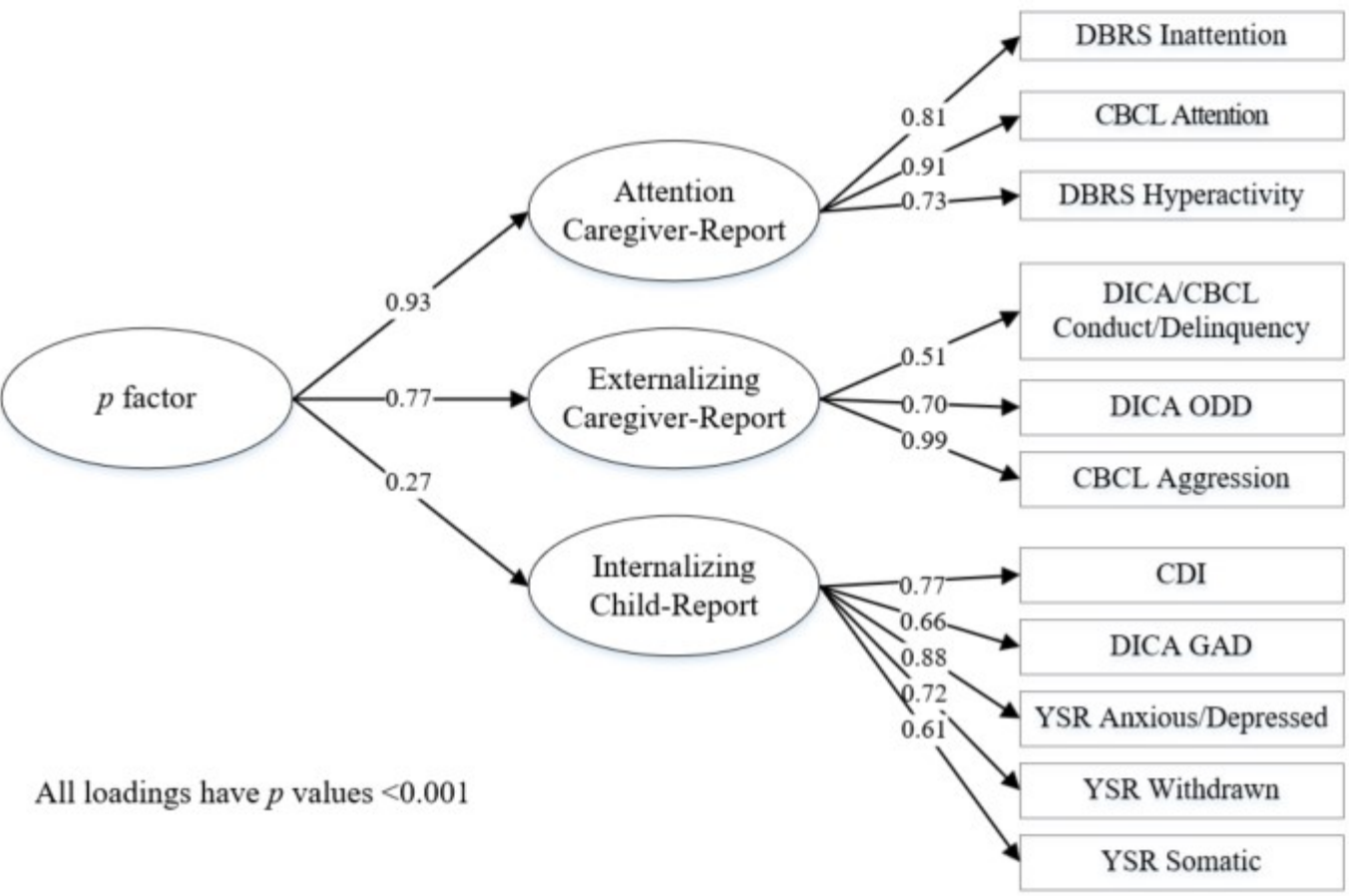

All loadings have *p* values <0.001

Supplement: Supp_1 [file NIHMS1912892-supplement-Supp_1.pdf]

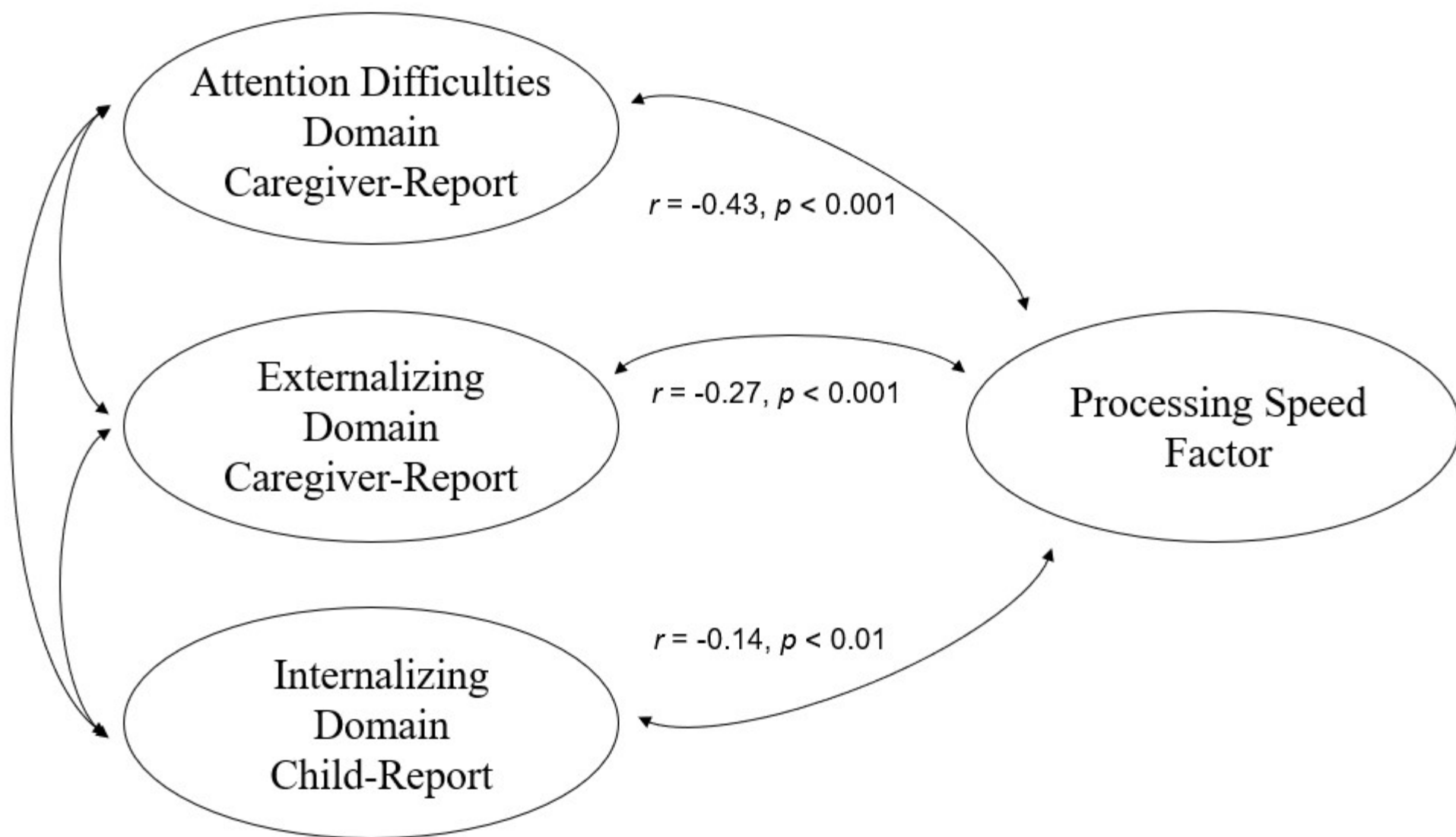

Supplement: Supp_2 [file NIHMS1912892-supplement-Supp_2.pdf]
