# Supplementary material for: Processing Speed is Related to the General Psychopathology Factor in Youth
Source: Res Child Adolesc Psychopathol. Author manuscript; Available in PMC 2024 Aug 1. (PMC10368543; doi:10.1007/s10802-023-01049-w)
Supplement: Supp_3 [file NIHMS1912892-supplement-Supp_3.docx]

**Supplementary Information:**

TITLE: Processing speed is related to the general psychopathology factor in youth

**Supplementary Information, Table 1: Descriptive statistics for psychopathology and processing speed measures**

|  | **N** | **Mean** | **SD** | **Range** |
| --- | --- | --- | --- | --- |
| **Measure** |  |  |  |  |
| ***Internalizing: Child Self-Report*** |  |  |  |  |
| CDI Total –  item count (27 items, 0-2 scale) | 787 | 4.96 | 5.20 | 0-34 |
| DICA GAD module –  symptom count (9 symptoms, yes/no) | 792 | 2.38 | 2.02 | 0-9 |
| YSR Anxious/depressed –  symptom count (16 items, 0-2 scale) | 698 | 4.17 | 4.07 | 0-24 |
| YSR Withdrawn–  item count (7 items, 0-2 scale) | 698 | 2.91 | 2.21 | 0-11 |
| YSR Somatic –  item count (9 items, 0-2 scale) | 698 | 2.63 | 2.58 | 0-15 |
| ***Externalizing: Caregiver-Report*** |  |  |  |  |
| ODD Interview –  symptom count (8 symptoms, yes/no) | 781 | 1.41 | 1.74 | 0-8 |
| Conduct/Delinquency scale –  symptom count (8 symptoms, yes/no and 13 symptoms, 0-2 scale) | 775 | 1.96 | 3.06 | 0-24 |
| CBCL Aggression –  item count (20 items, 0-2 scale) | 786 | 4.94 | 5.68 | 0-32 |
| ***Attention/Hyperactivity: Caregiver-Report*** |  |  |  |  |
| DBRS Inattention –  item count (9 items, 0-3 scale) | 790 | 7.21 | 6.31 | 0-27 |
| DBRS Hyperactivity/Impulsivity –  item count (9 items, 0-3 scale) | 790 | 4.06 | 4.64 | 0-25 |
| CBCL Inattention –  item count (11 items, 0-2 scale) | 787 | 2.71 | 3.41 | 0-19 |
| ***Processing Speed Measures*** |  |  |  |  |
| WISC-III/R Coding raw score | 792 | 55.93 | 13.72 | 18-93 |
| WISC-III Symbol Search raw score | 602 | 32.22 | 6.20 | 15-45 |
| Colorado Perceptual Speed Test raw score | 795 | 24.17 | 8.86 | 2-57 |
| Identical Pictures Test raw score | 795 | 68.92 | 14.52 | 0-96 |

CDI = Child Depression Inventory; DICA GAD = Diagnostic Interview for Children and Adolescents, Generalized Anxiety Disorder module; YSR = Youth Self Report; ODD = Oppositional Defiant Disorder; CBCL = Child Behavior Checklist; DBRS = Disruptive Behavior Rating Scale; WISC = Wechsler Intelligence Scale for Children

**Supplementary Information, Table 2: Model building: models including reports from caregiver 1 and caregiver 2 for externalizing symptoms, child self-report for internalizing symptoms**

| Model | *Χ^2^* | *df* | *p* | CFI | RMSEA  (90% CI) | AIC | BIC | SRMR |
| --- | --- | --- | --- | --- | --- | --- | --- | --- |
| 1.) 1 factor | 2342 | 119 | <0.001 | 0.54 | 0.15  (0.15-0.16) | 27933 | 28172 | 0.14 |
| 2.) 2 factor | 1309 | 118 | <0.001 | 0.75 | 0.11  (0.11-0.12) | 26603 | 26846 | 0.07 |
| 3.) 3 factor | 960 | 116 | <0.001 | 0.82 | 0.10  (0.09-0.10) | 26181 | 26434 | 0.07 |

**Supplementary Information, Table 3: Model fit statistics for substructure of the second-order *p* factor; main model with child self-report for internalizing measures and one caregiver report for externalizing and attention measures**

| **Model** | ***Χ^2^*** | ***df*** | ***p* value** | **CFI** | **RMSEA**  **(90% CI)** | **SRMR** | **AIC** | **BIC** | **Comparative test of nested models*** |
| --- | --- | --- | --- | --- | --- | --- | --- | --- | --- |
| 1a) 1 factor | 1496 | 44 | p < 0.001 | 0.50 | 0.20  (0.20-0.21) | 0.18 | 19854 | 20008 |  |
| 2a) 2 factors (internalizing and externalizing) | 385 | 43 | p < 0.001 | 0.88 | 0.10  (0.09-0.11) | 0.05 | 18534 | 18693 | 2a vs 1:  Δ*Χ^2^*(1)=634,  p < 0.001 |
| 2b) 2 factors, including a residual correlation between the two inattention scales | 215 | 42 | p < 0.001 | 0.94 | 0.07  (0.06-0.08) | 0.05 | 18333 | 18496 | 2b vs 2a:  Δ*Χ^2^*(1)=115,  p < 0.001 |
| **3) 3 factors** (internalizing, externalizing, and attention) | **153** | **41** | **p<0.001** | **0.96** | **0.06**  **(0.05-0.07)** | **0.04** | **18262** | **18430** | 2b vs 3:  Δ*Χ^2^*(1)=37,  p < 0.001 |

*These nested comparisons are calculated using the Satorra-Bentler scaled chi-square difference test (Satorra & Bentler, 2010), adapted for maximum likelihood estimation with robust standard errors (MLR).

**Supplementary Information, Figure 1: Standardized loadings for primary model: second-order, mixed-reporter model (child self-report for internalizing, caregiver-report for externalizing, attention)**

**
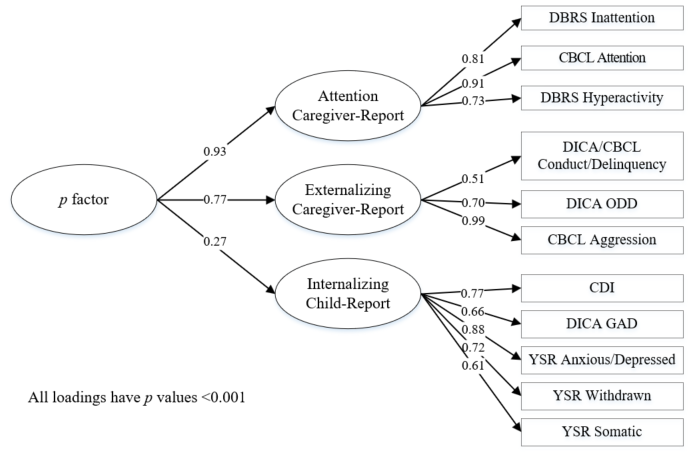
**

DBRS = Disruptive Behavior Rating Scale; CBCL = Child Behavior Checklist; ODD = Oppositional Defiant Disorder; CDI = Child Depression Inventory; DICA GAD = Diagnostic Interview for Children and Adolescents, Generalized Anxiety Disorder module; YSR = Youth Self Report

This figure indicates standardized loadings of the primary model (second-order, mixed-reported) before being correlated with processing speed. This model fit the data well (Χ^2^(41)=153, p<0.001, CFI=0.96, RMSEA=0.06 [90% CI 0.05-0.07], SRMR=0.04).

**Supplementary Information, Figure 2: Correlations between first-order latent factors and processing speed in a correlated traits model**


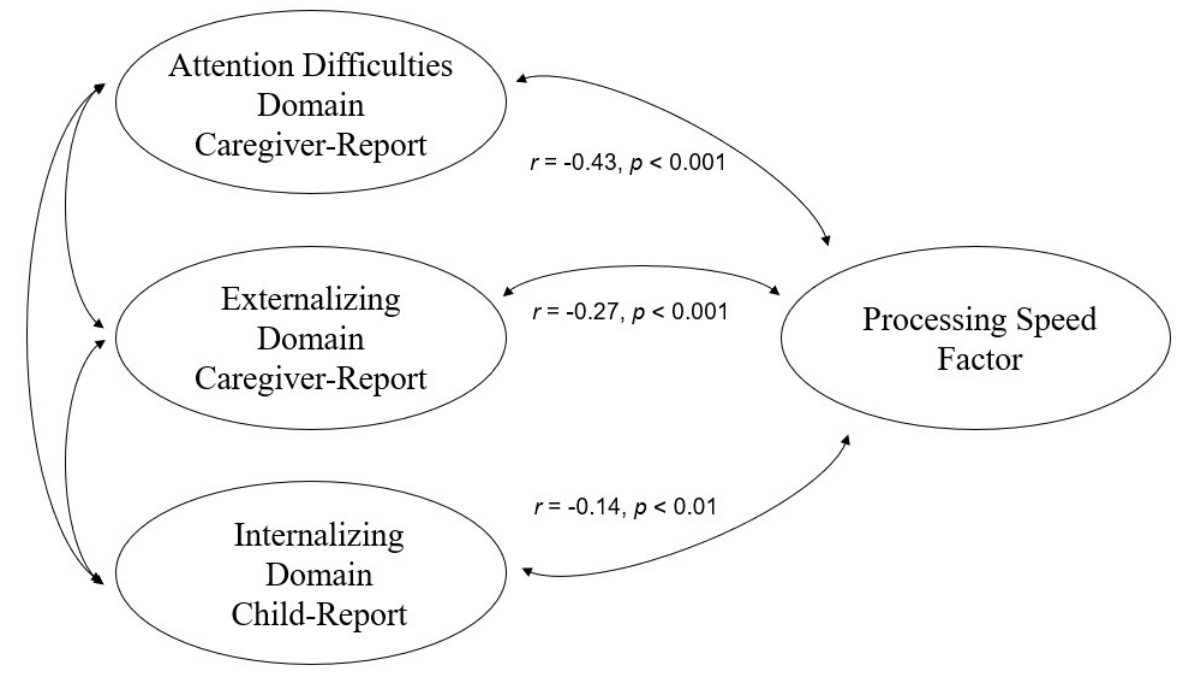


In the three-factor correlated traits model using the mixed-reporter method, the PS factor was significantly associated (*p*<0.01) with the three first-order factors (*r*=-0.14 with internalizing, -0.27 with externalizing, and -0.43 with attention; Figure S2). This model fit the data well (Χ^2^(84)=248, p<0.001, CFI=0.96, RMSEA=0.05 [90% CI 0.04-0.06], SRMR=0.04).

**Supplementary Information, Table 4: Model building for substructure of second-order model: correlated traits models with just one caregiver report for all symptoms (no child self-report)**

| Model | *Χ^2^* | *df* | *p* | CFI | RMSEA  (90% CI) | AIC | BIC | SRMR |
| --- | --- | --- | --- | --- | --- | --- | --- | --- |
| 1.) 1 factor | 794 | 44 | <0.001 | 0.72 | 0.15  (0.14-0.16) | 19497 | 19651 | 0.08 |
| 2.) 2 factor | 574 | 43 | <0.001 | 0.80 | 0.13  (0.12-0.14) | 19200 | 19359 | 0.07 |
| 3.) 3 factor | 337 | 41 | <0.001 | 0.89 | 0.10  (0.09-0.11) | 18888 | 19056 | 0.05 |

**Supplementary Information, Table 5: Standardized loadings for caregiver-report only, second-order model:**

|  | **Loading onto their domain-specific factor** |
| --- | --- |
| ***Caregiver report internalizing*** |  |
| DICA GAD module | 0.44*** |
| DICA MDD module | 0.58*** |
| CBCL Anxious/depressed | 0.87*** |
| CBCL Withdrawn | 0.71*** |
| CBCL Somatic | 0.51*** |
| ***Caregiver report externalizing*** |  |
| ODD Interview | 0.69*** |
| Conduct/Delinquency scale | 0.51*** |
| CBCL Aggression | 0.99*** |
| ***Caregiver report attention*** |  |
| DBRS Inattention | 0.80*** |
| DBRS Hyperactivity/Impulsivity | 0.70*** |
| CBCL Inattention | 0.93*** |
|  | **Loading of domain-specific factor onto the *p* factor** |
| Internalizing Domain | 0.80*** |
| Externalizing Domain | 0.79*** |
| Attention Domain | 0.89*** |
|  |  |
| **Model fit:** | Χ^2^(86)=468, CFI= 0.90, RMSEA = 0.08, SRMR = 0.05 |
| **Processing speed result for this model:** | Processing speed was significantly correlated with the *p* factor, *r*= -0.40, p<0.001 |

DICA GAD = Diagnostic Interview for Children and Adolescents, Generalized Anxiety Disorder module; MDD = Major Depressive Disorder; CBCL = Child Behavior Checklist; ODD = Oppositional Defiant Disorder; DBRS = Disruptive Behavior Rating Scale

*****p<0.05; **p<0.01; ***p<0.001

**Supplementary Information, Table 6: Standardized loadings for mixed-reporter (child self-report for internalizing, caregiver-report for externalizing, attention), bifactor model:**

|  | **Loading onto their domain-specific factor** | **Loading onto the *p* factor** |
| --- | --- | --- |
| ***Child report internalizing*** |  |  |
| CDI Total | 0.72*** | 0.26*** |
| DICA GAD module | 0.65*** | 0.13** |
| YSR Anxious/depressed | 0.87*** | 0.19*** |
| YSR Withdrawn | 0.71*** | 0.15*** |
| YSR Somatic | 0.57*** | 0.22*** |
| ***Caregiver report externalizing*** |  |  |
| ODD Interview | 0.55*** | 0.49*** |
| Conduct/Delinquency scale | 0.37*** | 0.40*** |
| CBCL Aggression | 0.63*** | 0.72*** |
| ***Caregiver report attention*** |  |  |
| DBRS Inattention | -- | 0.81*** |
| DBRS Hyperactivity/Impulsivity | -- | 0.72*** |
| CBCL Inattention | -- | 0.91*** |
|  |  |  |
| **Model fit:** | Χ^2^(81)=229, CFI= 0.96, RMSEA = 0.05, SRMR = 0.04 | |
| **Processing speed result for this model:** | Processing speed was significantly correlated with the *p* factor, *r*= -0.43, p<0.001 | |

CDI = Child Depression Inventory; DICA GAD = Diagnostic Interview for Children and Adolescents, Generalized Anxiety Disorder module; YSR = Youth Self Report; ODD = Oppositional Defiant Disorder; CBCL = Child Behavior Checklist; DBRS = Disruptive Behavior Rating Scale

*****p<0.05; **p<0.01; ***p<0.001

In the bifactor model, the attention factor did not have significant residual variance after adding the general factor into the model, which was expected given the very high loading of attention onto the second-order *p* factor. Thus, we loaded the attention indicators directly onto the general factor and did not have a domain-specific factor for attention.

PS was *not* significantly related to the domain-specific factors after accounting for the *p* factor (*r*(81)=-0.03, *p*=0.48 for internalizing, *r*(81)=0.06, *p*=0.14 for externalizing).

**Supplementary Information, Table 7: Standardized loadings for caregiver-report only, bifactor model**

|  | **Loading onto their domain-specific factor** | **Loading onto the *p* factor** |
| --- | --- | --- |
| ***Caregiver report internalizing*** |  |  |
| DICA GAD | 0.48*** | 0.21*** |
| DICA MDD module | 0.31*** | 0.47*** |
| CBCL Anxious/depressed | 0.65*** | 0.62*** |
| CBCL Withdrawn | 0.39** | 0.56*** |
| CBCL Somatic | 0.33*** | 0.37*** |
| ***Caregiver report externalizing*** |  |  |
| ODD Interview | 0.54*** | 0.50*** |
| Conduct/Delinquency scale | 0.36*** | 0.39*** |
| CBCL Aggression | 0.62*** | 0.73*** |
| ***Caregiver report attention*** |  |  |
| DBRS Inattention | -- | 0.80*** |
| DBRS Hyperactivity/Impulsivity | -- | 0.70*** |
| CBCL Inattention | -- | 0.92*** |
|  |  |  |
| **Model fit:** | Χ^2^(79)=456, CFI= 0.90, RMSEA = 0.08, SRMR = 0.05 | |
| **Processing speed result for this model:** | Processing speed was significantly correlated with the *p* factor, *r*= -0.42, p<0.001 | |

DICA GAD = Diagnostic Interview for Children and Adolescents, Generalized Anxiety Disorder module; MDD = Major Depressive Disorder; CBCL = Child Behavior Checklist; ODD = Oppositional Defiant Disorder; DBRS = Disruptive Behavior Rating Scale

*****p<0.05; **p<0.01; ***p<0.001

**Supplementary Information, Table 8: Omega estimates measuring reliability of the mixed-reporter, bifactor model and the caregiver-only report, bifactor model**

|  | **Mixed-reporter, bifactor model** | **Caregiver-only report, bifactor model** |
| --- | --- | --- |
| **Omega total proportion of variance** | ωt=0.90 | ωt=0.90 |
| **Omega hierarchical (*p* factor)** | ωh=0.56 | ωh=0.74 |
| **Omega hierarchical subscale: internalizing domain** | ωhs=0.79 | ωhs=0.42 |
| **Omega hierarchical subscale: externalizing domain** | ωhs=0.40 | ωhs=0.39 |

Given the concerns related to bifactor models, the current recommendation is to examine omega coefficients, which quantify reliability, strength, and dimensionality of the latent model (Forbes et al., 2021). In our mixed-reporter bifactor model, the total proportion of variance explained by the whole model was excellent (ωt=0.90), the proportion of variance explained by the general factor was acceptable but not ideal (ωh=0.56), and the proportion of variance explained by subdomains after accounting for the *p* factor were variable (ωhs=0.79 and 0.40 for internalizing and externalizing domains, respectively).

In the caregiver-only bifactor model, the general factor was more reliable than the mixed-reporter general factor (ωh=0.74), though the specific factors were similar or less reliable ((ωhs=0.42 and 0.39 for internalizing and externalizing domains, respectively).

**Supplementary Information, Table 9: Standardized loadings of the mixed-reporter, second-order model with no measures of attention or hyperactivity**

|  | **Loading onto their domain-specific factor** |
| --- | --- |
| ***Child report internalizing*** |  |
| CDI Total | 0.77*** |
| DICA GAD module | 0.66*** |
| YSR Anxious/depressed | 0.88*** |
| YSR Withdrawn | 0.72*** |
| YSR Somatic | 0.61*** |
| ***Caregiver report externalizing*** |  |
| ODD Interview | 0.70*** |
| Conduct/Delinquency scale | 0.52*** |
| CBCL Aggression | 0.98*** |
|  | **Loading of domain-specific factor onto the *p* factor** |
| Internalizing Domain | 0.33*** |
| Externalizing Domain | 0.64*** |
|  |  |
| **Model fit:** | Χ^2^(51)=104, CFI= 0.98, RMSEA = 0.04, SRMR = 0.03 |
| **Processing speed result for this model:** | Processing speed was significantly correlated with the *p* factor, *r*= -0.42, p<0.001 |

CDI = Child Depression Inventory; DICA GAD = Diagnostic Interview for Children and Adolescents, Generalized Anxiety Disorder module; YSR = Youth Self Report; ODD = Oppositional Defiant Disorder; CBCL = Child Behavior Checklist

*****p<0.05; **p<0.01; ***p<0.001

**Supplementary Information, Table 10: Standardized loadings of second-order, mixed-reporter model, accounting for general cognition (verbal and nonverbal reasoning tasks from the WISC)**

|  | **Loading onto their domain-specific factor** |
| --- | --- |
| ***Child report internalizing*** |  |
| CDI Total | 0.77*** |
| DICA GAD module | 0.66*** |
| YSR Anxious/depressed | 0.88*** |
| YSR Withdrawn | 0.72*** |
| YSR Somatic | 0.61*** |
| ***Caregiver report externalizing*** |  |
| ODD Interview | 0.70*** |
| Conduct/Delinquency scale | 0.51*** |
| CBCL Aggression | 0.99*** |
| ***Caregiver report attention*** |  |
| DBRS Inattention | 0.81*** |
| DBRS Hyperactivity/Impulsivity | 0.72*** |
| CBCL Inattention | 0.91*** |
|  | **Loading of domain-specific factor onto the *p* factor** |
| Internalizing Domain | 0.26*** |
| Externalizing Domain | 0.71*** |
| Attention Domain | 1.00*** |
|  | **Loadings of cognitive tasks onto the general cognition factor** |
| Block Design | 0.58*** |
| Picture Completion | 0.49*** |
| Picture Arrangement | 0.41*** |
| Object Assembly | 0.49*** |
| Information | 0.79*** |
| Similarities | 0.73*** |
| Vocabulary | 0.81*** |
| Comprehension | 0.65*** |
|  |  |
| **Model fit:** | Χ^2^(225) = 897, CFI = 0.90, RMSEA = 0.06, SRMR = 0.06 |

CDI = Child Depression Inventory; DICA GAD = Diagnostic Interview for Children and Adolescents, Generalized Anxiety Disorder module; YSR = Youth Self Report; ODD = Oppositional Defiant Disorder; CBCL = Child Behavior Checklist; DBRS = Disruptive Behavior Rating Scale

*****p<0.05; **p<0.01; ***p<0.001

**Supplementary Information, Table 11: Secondary analysis using raw scores for psychopathology and processing speed indicators, with age and sex as covariates**

| **Standardized beta coefficients of age and sex predicting psychopathology and PS** | | | |
| --- | --- | --- | --- |
|  | Age coefficient | Sex coefficient | Model |
| Child-report internalizing factor | *β* = 0.01, ns | *β* = 0.07, *p*=0.09 | Correlated traits |
| Caregiver-report externalizing factor | *β* = -0.02, ns | *β* = -0.10* | Correlated traits |
| Caregiver-report attention factor | *β* = -0.06, ns | *β* = -0.24*** | Correlated traits |
| Second-order *p* factor | *β* = -0.05, ns | *β* = -0.22*** | Second-order |
| Processing speed factor | *β* = 0.66*** | *β* = 0.19*** | Second-order |
|  | | | |
| **Correlations between psychopathology and PS in this model where age and sex are covariates** | | | |
|  | Correlation with PS | Model |  |
| Child-report internalizing factor | *r*(106)= -0.13** | Correlated traits |  |
| Caregiver-report externalizing factor | *r*(106) = -0.29*** | Correlated traits |  |
| Caregiver-report attention factor | *r*(106)= -0.44*** | Correlated traits |  |
| Second-order *p* factor | *r*(113)= -0.44*** | Second-order |  |

Note – positive values for the sex coefficient indicate females had higher levels of psychopathology or speed; negative values indicate males had higher levels of psychopathology or speed.

*****p<0.05; **p<0.01; ***p<0.001

We ran a secondary analysis using age and sex as covariates of latent PS and psychopathology to understand the influence of these variables on the relationship between psychopathology and PS (Table S12). Previous models, described above, had accounted for age and sex prior to model-building, while this analysis examined uncorrected raw scores. To do this, we used measures of raw psychopathology and processing speed, uncorrected for age and sex, as indicators in the structural equation model to create the correlated traits model and second-order, mixed-reporter *p* factor.

We regressed age and caregiver-identified sex onto the PS factor and the three correlated traits (internalizing, externalizing, and attention factors). Beta coefficients are reported in the Table S11. In line with previous PS literature (Camarata & Woodcock, 2006), age and sex were related to PS (older participants and females were faster on average than younger participants and males). Somewhat surprisingly, age was not related to any of the three psychopathology domains in the correlated traits model, possibly because of the restricted age range in this sample (11-16). Caregiver-identified sex was related to externalizing and inattention symptoms, with males having higher rates of these symptoms. Sex was not significantly related to internalizing symptoms.

We then created the second-order model to assess age and sex effects on the *p* factor. We regressed age and caregiver-identified sex onto the *p* factor and the PS factor. Age was not significantly related to the *p* factor. Caregiver-identified sex was related to the *p* factor, with males having higher general psychopathology. This was expected because males had higher rates of externalizing and inattention difficulties (described above) which compose two-thirds of the *p* factor for this study.

We then correlated the *p* factor and PS to assess if this different methodology for model-building changed the correlation between the two. The correlation between PS and psychopathology was similar (*r*(113)=-0.44) with this alternative model-building approach, providing good convergence with the primary models.
